# Using mimicry of body movements by a virtual agent to increase synchronization behavior and rapport in individuals with schizophrenia

**DOI:** 10.1038/s41598-018-35813-6

**Published:** 2018-11-26

**Authors:** Stéphane Raffard, Robin N. Salesse, Catherine Bortolon, Benoit G. Bardy, José Henriques, Ludovic Marin, Didier Stricker, Delphine Capdevielle

**Affiliations:** 1 University Department of Adult Psychiatry, Hôpital de la Colombière, CHU Montpellier, Montpellier, France; 2 Epsylon Laboratory EA 4556, Univ Paul Valéry Montpellier 3, Univ Montpellier, Montpellier, France; 3 EuroMov, Univ. Montpellier, Montpellier, France; 4 Institut Universitaire de France, Paris, France; 5 German Research Center for Artificial Intelligence (DFKI GmbH), University of Kaiserslautern, Kaiserslautern, Allemagne Germany; 6 INSERM U-1061, Montpellier, France

## Abstract

Synchronization of behavior such as gestures or postures is assumed to serve crucial functions in social interaction but has been poorly studied to date in schizophrenia. Using a virtual collaborative environment (VCS), we tested 1) whether synchronization of behavior, i.e., the spontaneous initiation of gestures that are congruent with those of an interaction partner, was impaired in individuals with schizophrenia compared with healthy participants; 2) whether mimicry of the patients’ body movements by the virtual interaction partner was associated with increased behavioral synchronization and rapport. 19 patients and 19 matched controls interacted with a virtual agent who either mimicked their head and torso movements with a delay varying randomly between 0.5 s and 4 s or did not mimic, and rated feelings of rapport toward the virtual agent after each condition. Both groups exhibited a higher and similar synchronization behavior of the virtual agent forearm movements when they were in the Mimicry condition rather than in the No-mimicry condition. In addition, both groups felt more comfortable with a mimicking virtual agent rather than a virtual agent not mimicking them suggesting that mimicry is able to increase rapport in individuals with schizophrenia. Our results suggest that schizophrenia cannot be considered anymore as a disorder of imitation, particularly as regards behavioral synchronization processes in social interaction contexts.

## Introduction

It is now well admitted that individuals have a tendency to synchronize their behaviors, which constitutes a key form of social exchange throughout the lifetime. Prototypical examples are found in everyday life when people are walking side by side and synchronize unconsciously their steps as well as their attitudes while talking to each other^1^. A substantial number of studies have shown that the foundation for this laid very early in development, as newborn infants spontaneously imitate a range of simple behaviors of an adult model^2^.

In addition to mimicry, which is typically defined as the spontaneous, immediate (at the split of a second) imitation of gestures, postures, mannerisms and the dynamics of movement of another person^3–5^, behavioral synchronization denotes the mutual alignment of interaction partners’ behavior on a larger time-scale. Both processes have been found to be critical in human-human interactions since they are suggested to be tied to liking, rapport and empathy^6^. For example, there is a large amount of evidence that being mimicked promotes rapport^7^, trust^8^, altruistic behavior and liking between interacting partners^9^ and, in turn increases behavioral synchronization and movements in the mimicked partner via a variety of psychological and neural mechanisms^10^.

In comparison to other types of deliberate, goal-directed, voluntary imitation such as social learning^11^ or imitation learning^12^, one crucial aspect of behavioral synchronization is the fact that it occurs without awareness. Indeed, behavioral synchronization is nonconscious, unintentional, effortless, and has the potency to increase prosocial behavior mostly when interacting people are not aware that their behavior is being mimicked. By contrast, overt detection of being mimicked can have deleterious effects on affiliation^13^ or liking^14^. In addition, as mimicry of other people’s behavior during social interaction is unconscious, it is thus fundamentally considered as a social behavior modulated by social cues such as eye gaze or social status^15^.

In the field of psychopathology and neurodevelopmental disorders, mimicry has been particularly studied in autism spectrum disorders. A recent meta-analysis^16^ indicated that individuals with autism exhibit significant impairments in behavioral synchronization skills on a variety of tasks including body movements, vocalizations, or facial expressions. The same results were found regarding mimicry^17,18^. Contrary to autism, behavioral synchronization and particularly its relationships with interpersonal functioning has been poorly studied in patients diagnosed with schizophrenia. As a whole, prior studies have repeatedly shown impaired voluntary imitation of face and complex hand gesture movements in schizophrenia patients in comparison to healthy partners^19–21^, especially when the production of a voluntary imitation depends upon working memory^22,23^. The few studies that focused on behavioral synchronization have produced mixed results. Whereas some studies showed impaired behavioral synchronization of emotional stimuli^24,25^, a recent study by Simonsen *et al*.^26^ failed to find any evidence of such impairment when controlling emotional and cognitive confounding variables in their group of patients diagnosed with schizophrenia.

Importantly, previous research in schizophrenia has mainly targeted nonsocial voluntary imitation or synchronization behavior of either complex or simple movements^21^ and employed experimental laboratory paradigms measuring specific body movements such as hands^22^, mouth^21^, or emotions expression^25,27,28^. In contrast, mimicry is traditionally studied during naturalistic paradigms that measure the frequency of mimicked movements during social interactions between a participant and a confederate (e.g. conversation).

Recent studies have found that automatic imitation^10^ or mimicry^29^ are independent of measures of empathy and social cognition. In line with these findings, reflective processes underlying social cognition (requiring effortful controlled processes) including metalizing, emotion regulation and perception of social stimuli (faces and voices), have been found to be systematically impaired in schizophrenia, while more reflexive aspects of social cognition (i.e. more automatic processes), such as motor resonance or emotion contagion, may be intact^30^. Therefore, we believe that mimicry (which is a reflexive aspect of social cognition) could constitute a promising tool for social rehabilitation in schizophrenia.

Recent advances in collaborative virtual environments (CVEs) have shown the potential of virtual reality for enhancing ecological validity while maintaining experimental control in social neuroscience research^31^. Indeed, contrary to a real human partner, virtual agents can be programmed to automatically mimic the participant’s movements after an accurate and fully controlled time delay. Importantly, a large amount of research has shown that individuals react towards virtual agents similarly than towards real people^32,33^. Accordingly, several studies have used CVEs to explore the positive effects of mimicry on interaction, and provided consistent results showing that mimicking virtual agents are perceived as more persuasive^34^, likable^35^, and trustworthy^36^.

For the reasons mentioned above, this study aimed to investigate 1) whether synchronization of behavior, i.e., the spontaneous initiation of gestures that are congruent with those of an interaction partner, was impaired in individuals with schizophrenia compared with healthy participants; 2) whether manipulating mimicry by a virtual agent would impact (a) synchronization behavior; (b) and the feeling of rapport associated with the virtual agent in two groups of nonclinical individuals and schizophrenia patients.

## Results

### Synchronization behavior

**Figure 1:**
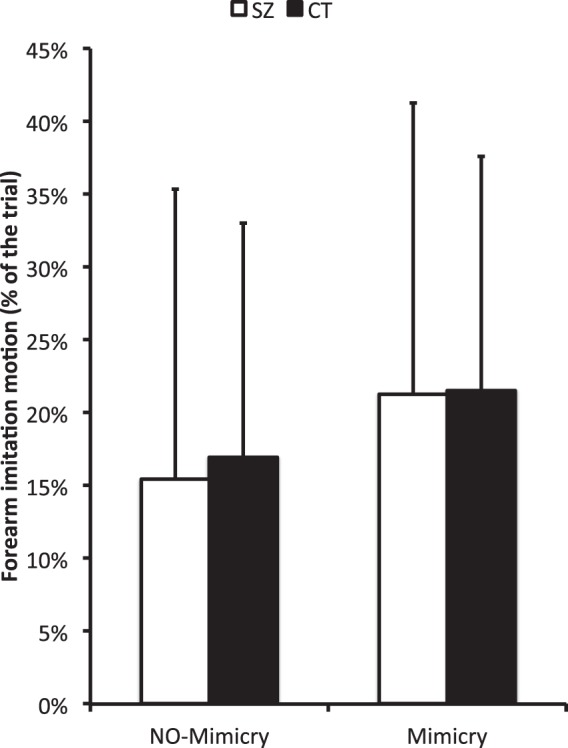
Mean percentage of the trials where a forearm imitation motion between virtual agent and participant is detected. Left columns refer to the no-mimicry conditions and right columns refer to the mimicry condition. Indicative information is provided by the colors where white columns refer to the schizophrenia group whereas black columns correspond to the control group. Error bars correspond to the standard deviation around the mean.

Results revealed a significant effect of mimicry (F(1,36) = 4.69, *p* = 0.037, *η*^2^_p_ = 0.12). This result displayed on Fig. 1 indicates that participants exhibited a higher synchronization behavior of the virtual agent forearm movements when they were in the Mimicry condition (21.4%) rather than in the No-mimicry condition (16.2%). The analysis failed to reveal any group difference (Tables 1 and 2).

**Table 1:** Descriptive statistics of the Mimicry, Amount and Rapport variables.

	Schizophrenia Patients (N = 19)				Healthy Controls (N = 19)			
	M	SD	Med	Rg	M	SD	Med	Rg
**Imitation**								
NO-Mimicry	15.4%	19.9%	9.2%	[0.3% 75.7%]	16.9%	16.1%	17.2%	[0.4% 52.2%]
Mimicry	21.3%	20.0%	19.3%	[0.1% 68.6%]	21.5%	16.0%	23.9%	[0.4% 62.3%]
**Amount (10e3)**								
NO-Mimicry	50.2	55.2	32.5	[1.1 165.4]	65.4	46.7	56.4	[8.8 146.1]
Mimicry	71.2	60.1	41.8	[3.4 218.5]	94.7	68.7	105.8	[0.0 220.5]
**Rapport**								
**Q1**								
NO-Mimicry	0.84	1.68	1.00	[−2 3]	1.32	1.16	1.00	[−1 3]
Mimicry	1.42	1.07	1.00	[0 3]	1.68	1.00	2.00	[−1 3]
**Q2**								
NO-Mimicry	0.63	1.34	0.00	[−2 3]	0.11	1.59	0.00	[−3 3]
Mimicry	0.47	1.65	1.00	[−3 3]	0.16	1.71	0.00	[−3 3]
**Q3**								
NO-Mimicry	0.58	1.43	1.00	[−2 3]	1.32	0.95	1.00	[0 3]
Mimicry	0.79	1.36	1.00	[−2 3]	1.00	1.00	1.00	[−1 3]
Q4								
NO-Mimicry	0.58	1.77	0.00	[−3 3]	0.68	1.11	1.00	[−1 3]
Mimicry	0.95	1.43	1.00	[−1 3]	1.05	1.78	1.00	[−1 3]

Mean, Standard deviation, Median and Range of values are presented for the schizophrenia group and the healthy control group in both Mimicry and NO-Mimicry conditions.

Q1: “I felt comfortable while interacting with this virtual agent”.

Q2: “I think this virtual agent is attractive”.

Q3: “I like this agent”.

Q4: “I want to interact with this virtual agent again in the future”.

**Table 2:** ANOVAs for the Mimicry, Range and Rapport variables. Group, Mimicry and Group × Mimicry effects are reported.

	Group	Mimicry	Group × Mimicry
Imitation	F(1,36) = 0.03, *p* = 0.87, η^2^_p_ = 0.00	**F(1,36) = 4.69**, ***p* = 0.037, η**^**2**^_**p**_** = 0.12**	F(1,36) = 0.07, *p* = 0.79, η^2^_p_ = 0.00
Amount (x10e3)	F(1,36) = 1.41, *p* = 0.24, η^2^_p_ = 0.04	**F(1,36) = 7.02**, ***p* = 0.012, η**^**2**^_**p**_** = 0.16**	F(1,36) = 0.19, *p* = 0.66, η^2^_p_ = 0.00
**Rapport**			
Q1	F(1,36) = 1.03, *p* = 0.32, η^2^_p_ = 0.03	**F(1,36) = 6.52**, ***p* = 0.015, η**^**2**^_**p**_** = 0.15**	F(1,36) = 0.32, *p* = 0.57, η^2^_p_ = 0.01
Q2	F(1,36) = 0.74, *p* = 0.39, η^2^_p_ = 0.02	F(1,36) = 0.11, *p* = 0.74, η^2^_p_ = 0.00	**F(1,36) = 0.45**, ***p* = 0.51, η**^**2**^_**p**_** = 0.01**
Q3	F(1,36) = 1.63, *p* = 0.21, η^2^_p_ = 0.04	F(1,36) = 0.20, *p* = 0.66, η^2^_p_ = 0.01	F(1,36) = 4.92, *p* = 0.033, η^2^_p_ = 0.12
Q4	F(1,36) = 0.21, *p* = 0.80, η^2^_p_ = 0.09	F(1,36) = 3.51, *p* = 0.069, η^2^_p_ = 0.09	F(1,36) = 0.00, *p* = 1, η^2^_p_ = 0.00

Q1: I felt comfortable while interacting with this virtual agent.

Q2: I think this virtual agent is attractive.

Q3: I like this agent.

Q4: I want to interact with this virtual agent again in the future.

### Amount of non-verbal behavior

The results of the ANOVA Groups (2) × Mimicry (2) performed on the range of motion of the left forearm revealed a significant Mimicry effect (F(1,36) = 7.02, *p* = 0.012, *η*^2^_p_ = 0.16) indicating that both groups exhibited larger arms movement when facing an mimicking virtual agent rather than an virtual agent not mimicking them (respectively 0.083 vs. 0.058 in arbitrary units). In other words, mimicry increased the amount of nonverbal behavior (i.e. movements) in both groups (Tables 1 and 2).

### Rapport

Regarding the questions evaluating how much participants felt comfortable during the interaction, results demonstrated a statistically significant effect of mimicry for one of the three questions (F(1,36) = 6.52, *p* = 0.015, *η*^2^_p_ = 0.15) indicating that both groups felt more comfortable with a mimicking virtual agent rather than a virtual agent not mimicking them (respectively +1.55 vs. +1.08). For the question associated with willingness to interact with the virtual agent in the future, no main effect was found statistically significant (Tables 1 and 2). A significant Group × Mimicry interaction was found significant for likeness (F(1,36) = 4.92, *p = 0.033*, *η*^2^_p_ = 0.12), however the post-hoc decomposition of this interaction failed to reveal any significant difference between the conditions. This is due to the between/within design of this interaction and therefore prevents any interpretation of such result. We also collapsed the four items to obtain a rapport total score, that is, rapport, attractiveness, likeness, and willingness to interact. Rapport total score also failed to found statistical differences (Group effect F(1,36) = 0.14, *p* = 0.71, *η*^2^_p_ = 0.004; Mimicry effect F(1,36) = 3.74, *p* = 0.06, *η*^2^_p_ = 0.094; Group × Mimicry interaction F(1,36) = 0.48, *p* = 0.49, *η*^2^_p_ = 0.013), however, we note here a statistical tendency (*p* = 0.06) with a small to medium effect size (*η*^2^_p_ = 0.094) for the mimicry condition.

### Mimicry, rapport and psychotic symptomatology

Regarding psychotic dimensions, range of motion in the Mimicry condition negatively correlated with the positive dimension of the five-factor model of the PANSS (r = −0.57, *p* = 0.034) and the perceived attractiveness (Q2) in the Mimicry condition positively correlates with the CPZ equivalent dose (r = 0.58, *p* = 0.029). No other significant correlations were found between Mimicry, rapport, psychotic symptomatology, and other clinical and demographic variables. After applying Bonferroni corrections for multiple comparisons with an alpha value of 0.05, no significant correlations remained statistically significant (Table 4).

**Table 3:** Socio-demographic description of the schizophrenia group and the healthy control group.

	Schizophrenia Patients (N = 19)				Healthy Controls (N = 19)				Statistics		
	M	SD	Med	Rg	M	SD	Med	Rg			
Age	31.9	9.4	30	22–59	30.5	8.4	27	21–47	*U* = 143.5	*z* = −0.30	*p* = 0.77
Level of Education	13.0	2.6	13	9–17	13.7	2.2	14	9–17	*U* = 143.5	*z* = 1.07	*p* = 0.28
fNART	25.1	6.1	27	13–33	27.8	4.3	29	18–34	*U* = 135.5	*z* = 1.30	*p* = 0.19
PANSS Positive	12.5	4.2	14	7–19							
PANSS Negative	13.1	3.8	13	8–20							
PANSS General Psychopathology	24.5	6.2	23	17–41							
PANSS Total score	50.1	10.4	51	34–76							
PANSS-5-POSITIVE	13.1	5.8	14	5–25							
PANSS-5-NEGATIVE	13.4	4.5	13	6–21							
PANSS-5-DISORGANIZATION	13.3	2.8	12	10–20							
PANSS-5-EXCITMENT	11.2	2.3	11	8–16							
PANSS-5-EMOTIONAL DISTRESS	16.0	5.9	16	8–27							
CPZ equivalent dose in mg	473.89	346.26	310	100–1500							
Illness Duration	9.06	7.3	6.5	2–31							
	**N**	**%**			**N**	**%**					
Gender/*Man*	16	84			17	89			*χ2* (1, N = 38) = 0.23	*p* = 0.63	

Mean, Standard deviation, Median and Range of values are presented for the Age, Gender, Level of education, general and sub dimensions of the PANSS, Illness duration and Chlorpromazine equivalent dose. Group statistics are presented using appropriates non-parametric tests.

**Table 4:** Correlation between motion/rapport variables and sociodemographic/clinical variables.

					Gender	Age	Level of Education	fNART	PANSS 5 Factors					PANSS				Illness Duration	CPZ equivalent dose
									Positive	Negative	Disorgan-ization	Excitement	Emotional Distress	Positive	Negative	General Psychop-athology	Total Score		
Amount of non-verbal behavior		NO-Mimicry	1	r	−,3594	−,1895	−,2030	,0303	,3293	,0453	,2761	,3119	,1631	,2073	,0049	,3172	,2783	−,0897	−,3548
				*p*	*p* = *=,207*	*p* = *=,516*	*p* = *=,487*	*p* = *=,918*	*p* = *=,250*	*p* = *=,878*	*p* = *=,339*	*p* = *=,278*	*p* = *=,577*	*p* = *=,477*	*p* = *=,987*	*p* = *=,269*	*p* = *=,335*	*p* = *=,760*	*p* = *=,213*
		Mimicry	2	r	−,1533	−,0950	−,1260	,1496	**−,5687**	−,1259	−,2675	−,3707	−,4071	**−,6182**	−,1811	−,3421	−,5294	,0195	,0200
				*p*	*p* = *=,601*	*p* = *=,747*	*p* = *=,668*	*p* = *=,610*	***p*** = ***=,034***	*p* = *=,668*	*p* = *=,355*	*p* = *=,192*	*p = =,149*	***p*** = ***=,018***	*p* = *=,536*	*p* = *=,231*	*p = =,052*	*p* = *=,947*	*p* = *=,946*
Imitation		NO-Mimicry	3	r	−,2536	−,0738	,0300	−,1404	,1542	−,1158	−,0431	−,0832	,2670	−,0381	−,2956	,2912	,0571	−,2330	−,2131
				*p*	*p = =,382*	*p* = *=,802*	*p* = *=,919*	*p* = *=,632*	*p* = *=,599*	*p* = *=,693*	*p* = *=,884*	*p* = *=,777*	*p* = *=,356*	*p* = *=,897*	*p* = *=,305*	*p* = *=,312*	*p* = *=,846*	*p* = *=,423*	*p* = *=,465*
		Mimicry	4	r	,0070	,0116	−,3385	−,0569	−,2713	−,2602	−,0412	−,4123	−,0951	−,3466	−,3506	,0136	−,2583	,0226	,2084
				*p*	*p* = *=,981*	*p* = *=,969*	*p* = *=,236*	*p* = *=,847*	*p* = *=,348*	*p* = *=,369*	*p* = *=,889*	*p* = *=,143*	*p* = *=,746*	*p* = *=,225*	*p* = *=,219*	*p* = *=,963*	*p* = *=,373*	*p* = *=,939*	*p* = *=,475*
Rapport	Q1	NO-Mimicry	5	r	,2079	,0573	−,2672	−,3889	−,0292	−,2277	,3837	−,1204	,0885	,0559	−,0209	,2059	,1385	−,0047	,4633
				*p*	*p* = *=,476*	*p* = *=,846*	*p* = *=,356*	*p* = *=,169*	*p* = *=,921*	*p = =,434*	*p* = *=,176*	*p* = *=,682*	*p = =,764*	*p* = *=,849*	*p* = *=,943*	*p* = *=,480*	*p* = *=,637*	*p* = *=,987*	*p* = *=,095*
		Mimicry	6	r	,2094	−,1349	−,1982	−,4496	−,0375	−,3920	,4588	−,1029	−,1158	,2062	−,0921	−,0431	,0336	−,1834	,3968
				*p*	*p* = *=,473*	*p* = *=,646*	*p* = *=,497*	*p* = *=,107*	*p* = *=,899*	*p* = *=,166*	*p* = *=,099*	*p* = *=,726*	*p* = *=,693*	*p* = *=,479*	*p* = *=,754*	*p* = *=,884*	*p* = *=,909*	*p* = *=,530*	*p* = *=,160*
	Q2	NO-Mimicry	7	r	,0189	−,3597	−,0704	−,1810	−,1710	−,1753	−,0512	−,0510	−,0207	−,1595	−,1692	,0118	−,1182	−,4058	,1091
				*p*	*p* = *=,949*	*p* = *=,207*	*p* = *=,811*	*p* = *=,536*	*p* = *=,559*	*p* = *=,549*	*p* = *=,862*	*p = =,863*	*p* = *=,944*	*p* = *=,586*	*p* = *=,563*	*p* = *=,968*	*p* = *=,687*	*p* = *=,150*	*p* = *=,710*
		Mimicry	8	r	,4327	−,0882	−,4256	−,4390	−,4176	−,2105	,3218	−,3509	−,4277	−,1430	,1330	−,3221	−,2077	−,0119	**,5820**
				*p*	*p* = *=,122*	*p* = *=,764*	*p* = *=,129*	*p* = *=,116*	*p* = *=,137*	*p* = *=,470*	*p* = *=,262*	*p* = *=,219*	*p* = *=,127*	*p* = *=,626*	*p* = *=,650*	*p* = *=,261*	*p* = *=,476*	*p* = *=,968*	***p*** = ***=,029***
	Q3	NO-Mimicry	9	r	,2607	−,2470	−,1313	−,3036	−,0642	−,2161	,1840	−,0294	−,0079	,0459	−,0854	,0289	,0087	−,2981	,2703
				*p*	*p* = *=,368*	*p* = *=,395*	*p* = *=,655*	*p* = *=,291*	*p* = *=,827*	*p* = *=,458*	*p* = *=,529*	*p* = *=,921*	p = =,979	*p* = *=,876*	*p* = *=,772*	*p* = *=,922*	*p* = *=,976*	*p* = *=,301*	*p* = *=,350*
		Mimicry	10	r	,3017	−,1507	−,0750	−,3081	−,0555	−,1992	,3123	,0544	−,1505	,1978	,0892	−,1389	,0335	−,1625	,2759
				*p*	*p* = *=,295*	*p* = *=,607*	*p* = *=,799*	*p* = *=,284*	*p* = *=,851*	*p* = *=,495*	*p* = *=,277*	p = =,854	p = =,608	*p* = *=,498*	*p* = *=,762*	*p* = *=,636*	*p* = *=,909*	*p* = *=,579*	*p* = *=,340*
	Q4	NO-Mimicry	11	r	,3585	−,1226	−,4090	−,4205	−,3000	−,2238	,3041	−,1514	−,3162	−,0534	,0722	−,2118	−,1239	−,0972	,4189
				*p*	*p* = *=,208*	*p* = *=,676*	*p* = *=,146*	*p* = *=,134*	*p* = *=,297*	*p* = *=,442*	p = =,291	p = =,605	p = =,271	*p* = *=,856*	*p* = *=,806*	*p* = *=,467*	*p* = *=,673*	*p* = *=,741*	*p* = *=,136*
		Mimicry	12	r	,3169	−,1420	−,0122	−,4187	,0437	−,2902	,3079	−,0856	−,0569	,2532	−,0479	−,0664	,0549	−,2365	,2536
				*p*	*p* = *=,270*	*p = =,628*	*p* = *=,967*	*p* = *=,136*	*p* = *=,882*	p = =,314	p = =,284	p = =,771	p = =,847	*p* = *=,382*	*p* = *=,871*	*p* = *=,822*	*p* = *=,852*	*p* = *=,416*	*p* = *=,382*

## Discussion

Our study investigated synchronization behavior during an interaction with a virtual agent in individuals with schizophrenia.

We programmed a virtual agent to mimic participants’ head and torso movements with a delay varying randomly between 0.5 s to 4 s, compared to a non-mimicry condition. We measured synchronization behavior of forearm movements, responses to being mimicked (i.e. rapport) and amount of non-verbal behaviors, between the two conditions.

First, our findings revealed that schizophrenia patients displayed intact synchronization behavior during interaction with virtual agents compared to the healthy controls group. Indeed, the two groups of participants exhibited similar and a higher synchronization behavior of the virtual agent forearm movements when they were in the mimicry condition rather than in the non-mimicry condition. In addition, both groups exhibited larger arms movement when facing a mimicking virtual agent rather than a virtual agent not mimicking them. On the one hand, this result appears in line with the recent study by Simonsen *et al*.^26^ who showed that their schizophrenia group displayed intact automatic imitation compared to matched healthy individuals. On the other hand our study contrasts with the majority of previous findings showing impaired imitation abilities in schizophrenia. First, and as stated by Simonsen *et al*.^26^, previous findings assessed voluntary imitation^21–23^ or automatic imitation of emotional stimuli^24,25^ without controlling for the cognitive, motoric, and/or emotional deficits associated with schizophrenia. Thus it makes difficult to conclude whether imitation deficits were primary or secondary to aforementioned other deficits. Second, our experiment focused on the behavioral synchronization with a virtual agent on a larger time-scale, whereas Simonen *et al*.^26^ examined automatic imitation of finger movements presented via short video sequences. Consequently, our findings indicate that the fundamental tendency to synchronize with the behavior of others in social interactions is preserved in individuals with schizophrenia. Additionally, our finding are in line with previous studies in nonclinical individuals^34,35^ showing that virtual agents have the potential to induce behavioral synchronization and mimicry during interactive paradigms and extends this result to individuals with schizophrenia disorder. Our results also fit well with the results of Riehle & Lincoln^37^. In their study the authors assessed the amount of smiling and mimicry of smiling via electromyography during a dyadic interaction in participants with schizophrenia and healthy controls. Similarly with our results, they did not find evidence of reduced mimicry of smiling in a face-to-face interaction in the patients group. Their results are particularly relevant with regards to our study. As contrary to our experimental task consisting of an interaction with a virtual agent, they used an ecological task (brief face-to-face conversation) during a human-human interaction. Taken together, these findings suggest that behavioral synchronization during social interaction is preserved in schizophrenia patients”.

Secondly and as hypothesized, our results indicated that behavioral synchronization was associated with better rapport in our two groups. Indeed, both groups felt more comfortable with a virtual agent that mimicked their behavior than the one that did not. In addition and regarding rapport, we found a marginally significant effect in the mimicry condition when we collapsed the four measures of rapport used in our study. This is consistent with previous research using virtual agents and mimicry that showed that mimicking virtual agents are perceived as more likable^35^ and trustworthy^36^. In a therapeutic context it may be translated in patients being more willing and feeling more comfortable to share personal information. Previous studies have shown that individuals evaluate therapeutic alliance with a virtual agent in a positive manner and are willing to share their personal information^38,39^. Associating mimicry and the use of virtual agents controlled by artificial intelligence or algorithm or virtual agents controlled by therapist/clinician may be a promising option to increase access and engagement to psychological services^40^.

It must be however noted that we did not find specific effects of mimicry on any of our other dependent measures (attractiveness, likeness, willingness to interact) suggesting that these measures of rapport and connectedness could not be adequate in the context of virtual environment. A validated scale of rapport in collaborative virtual environments could be particularly useful for future studies. It is worth mentioning that ratings of liking, and self-other overlap also show inconsistent effects of mimicry in both virtual environment and traditional research settings, leading some researchers to suggest that the positive social effect of being mimicked is perhaps either not always consciously perceived or more fragile than generally reported in the scientific literature^41^. Finally, it should be noted that the key findings of this study showed remarkable effect sizes, suggesting that they may be relevant beyond mere academic interest.

There is evidence that people imitate the behaviors of those with whom they have ongoing relationships: parents^42^ and teachers^43^. There is also evidence that higher coordination of patient’s and therapist’s movement in psychotherapy dyads is associated with better outcome^44^. However, mimicry occurs spontaneously and unintentionally and it is very difficult to voluntary imitate other’s body posture, hand gestures or emotions during an interaction without being aware that the behavior is being copied^45^. As a consequence, using virtual agents of well-known person (i.e. referent from a clinical team) could constitute a promising augmentation to virtual reality social skills training intervention or job training interview, via the use of mimicking virtual agents. In addition, series of previous studies have shown that social cues, such as eye-contact^17^, and emotional facial expressions^46^, have the potential to modulate synchronization behavior in healthy participants. Further studies are needed to explore if such additional social cues implemented in virtual partners could increase synchronization behavior and by consequences act as a “social” cue in a mental disorder with severe social impairments and rejection.

## Conclusion

First, our findings suggest that schizophrenia cannot be considered anymore as a disorder of imitation, particularly as regards behavioral synchronization processes in social interaction contexts. Secondly, our study demonstrated how virtual environments could be used to address important questions in the context of social disorders such as schizophrenia. From a clinical perspective, we believe that our results are very encouraging and have implications for VR training programs. Virtual reality environments have been increasingly used in the context of mental health treatment and within schizophrenia research during the last five years. Virtual reality, as a treatment approach, has demonstrated promise in treating psychotic symptoms particularly hallucination^47^, but also as an adjunct to social-skills training^48^ or job interview training^49^. As such, future virtual reality interventions could use mimicry as a real-time feedback signal to create more meaningful human-virtual agent interactions leading to increase patients’ engagement in VR-based treatment.

## Methods

### Participants

Nineteen outpatients with schizophrenia and nineteen healthy controls participated in the study. Two additional schizophrenia patients and two healthy participants did not complete the whole experiment. We recruited patients meeting DSM IV-TR criteria for schizophrenia in Montpellier University Hospital. Diagnosis of schizophrenia was established via the Structured Clinical Interview for DSM-IV (SCID-I) by the treating psychiatrist. None were in the acute phase of psychosis. Inclusion criteria were being between 18 and 55 years of age, having a diagnosis of schizophrenia and being able to understand, talk and read French. Exclusion criteria were substance dependency other than cannabis or tobacco, substance abuse other than cannabis or alcohol, and co-morbid neurological disorder. Eighteen patients were medicated at clinically determined dosages. One patient was not taking medication. The mean dose of antipsychotic medication was equivalent to 540 mg/day of chlorpromazine (SD = 334.38)^50^. Controls were recruited from the general population with no personal lifetime history of any psychosis or affective disorders diagnosis (MINI)^51^. Controls with a family member with bipolar or schizophrenia disorders were excluded. All participants were native French speakers with a minimal reading level validated using the National Adult Reading Test (f-NART)^52^. All participants were right-handed as assessed by informal verbal inquiry. All participants provided written informed consent, prior to the experiment approved by the National Ethics Committee (CPP Sud-Méditerranée-III, Nîmes, France, #2009.07.03ter and ID-RCB-2009-A00513-54) and conforming to the Declaration of Helsinki. Accordingly with identifying information policies, written informed consent for publication of identifying information/images was obtained.

### Clinical variables

In the schizophrenia sample, we administered the Positive and Negative Syndrome Scale using the Structured Clinical Interview for the PANSS (SCI-PANSS). For this study, five of the analytically derived PANSS factor component scores^53^ were taken into account: Positive, Negative, Disorganization, Excitement and Emotional Distress (Table 3).

### Rapport

No validated measures of rapport such as the Inclusion of the Other in the Self Scale (IOS)^54^ or the 6-item Willingness to Interact Questionnaire (WIQ)^55^ have been developed for CVEs. Thus, rapport during the interaction with the virtual agent was evaluated using self-reported measures based on previous studies using CVEs^56^ and on literature on interpersonal communication. The questionnaire was composed by three questions assessing positive rapport toward the virtual agent (i.e. “I felt comfortable while interacting with this virtual agent”, “I think this virtual agent is attractive”, “I like this agent”) and one question assessing willingness for future interactions adapted from the WIQ (“I want to interact with this virtual agent again in the future”). The questions were answered using a scale from −3 to +3, −3 being equals to “I do not agree al all”, 0 “more or less”, and +3 “I totally agree”. A rapport total score was also provided composed by the sum of the four questions.

### Procedure

**Figure 2:**
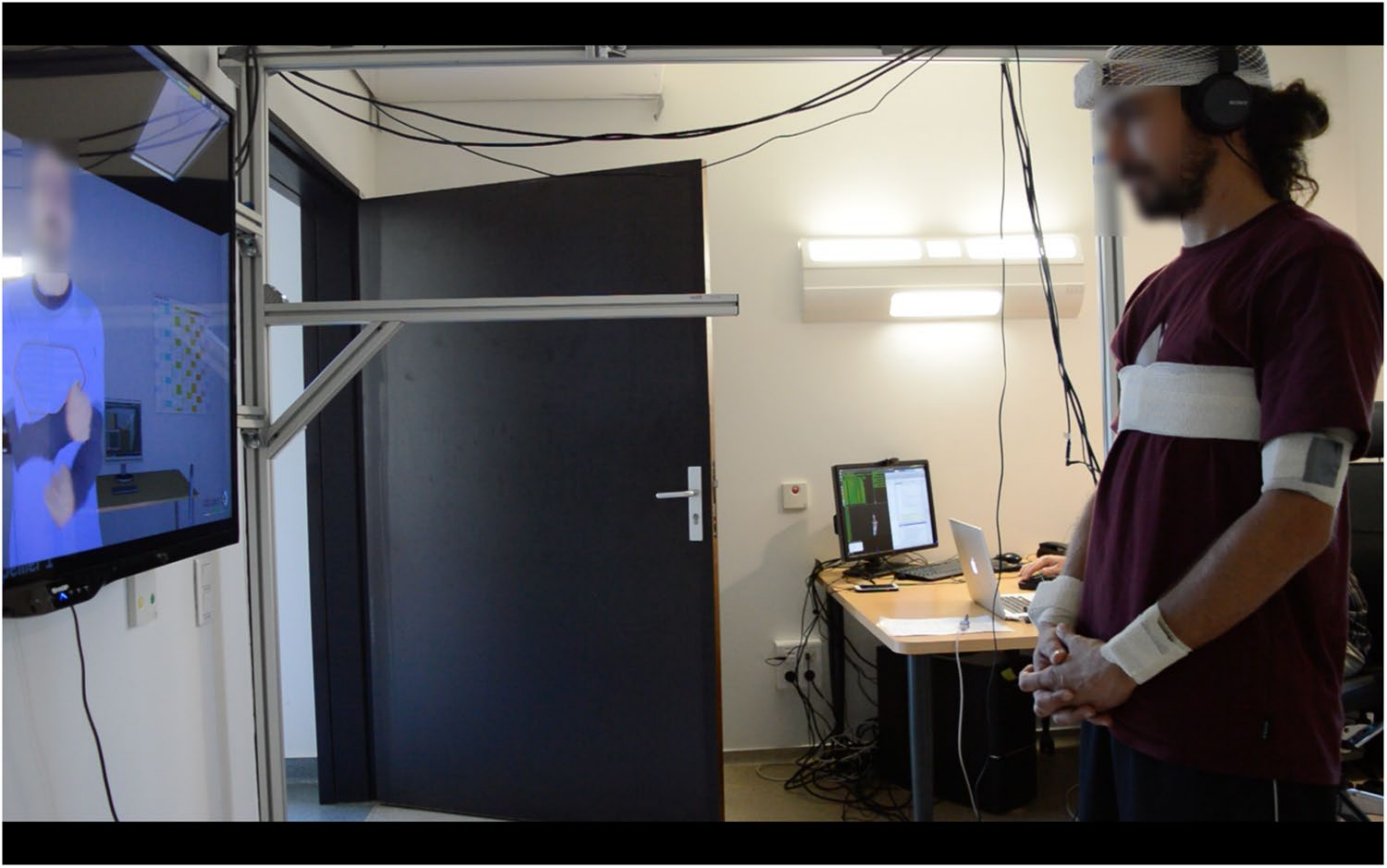
Picture of the interactive apparatus where participants stood up in front a photo-realistic 3D virtual agent displayed on a large TV screen. Sensors (IMUs) used to capture the motion can be seen on the head, torso, right arm and right forearm of the participant.

**Figure 3:**
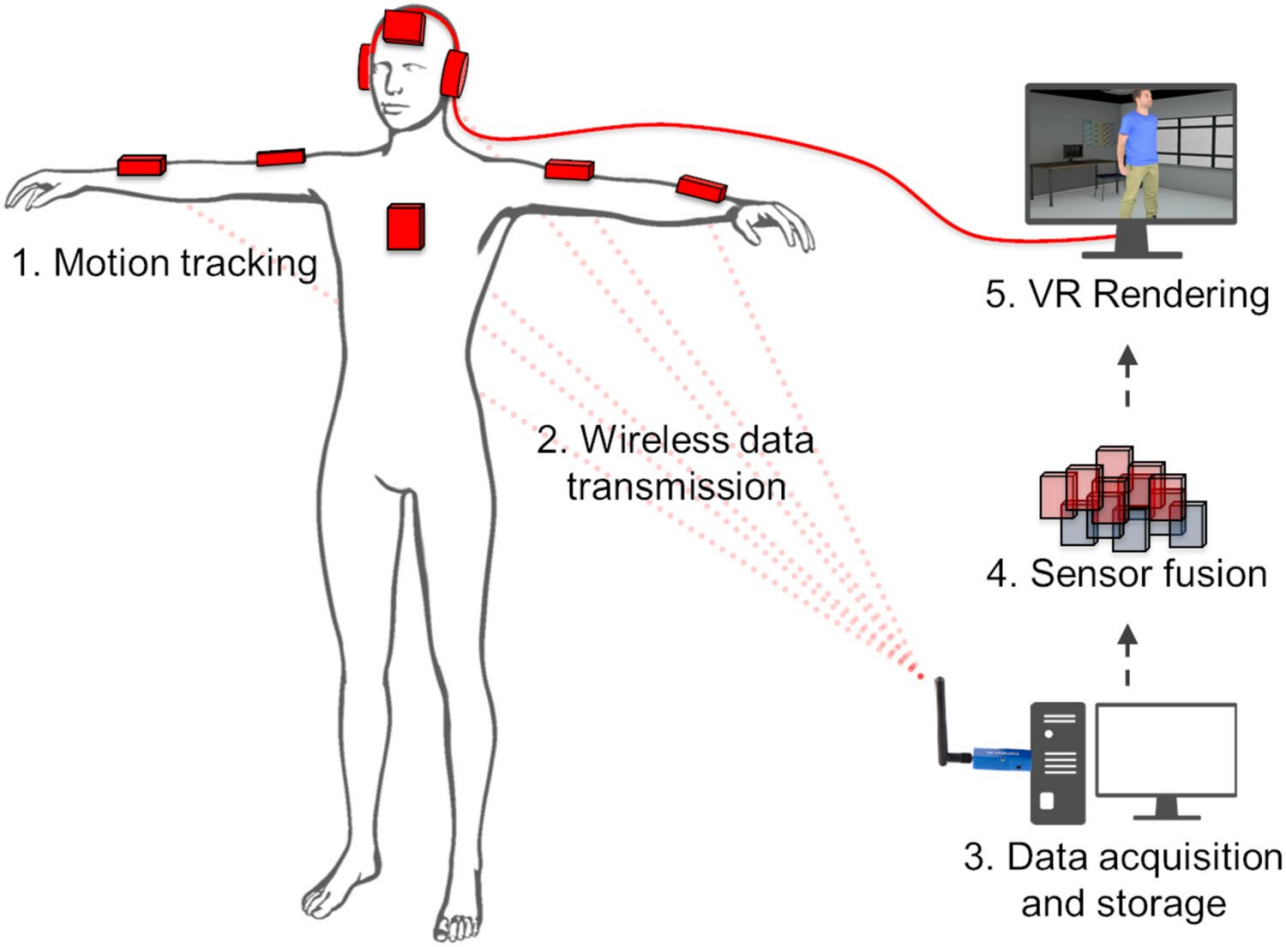
Schematic illustration of the Imitative Virtual Reality Pipeline used in this experiment. 1. Participant motion is tracked using 6 IMUs (red cuboids); 2. Data are wireless transmitted in real-time to the computer; 3. Data are stored and also used for further computation; 4. Data from the different sensors are fused as a function of the mimicry condition, in the no-mimicry condition, only prerecorded motions (blue cuboids) are sent to the rendering process whereas in the mimicry condition, head and torso motions of the participant (red cuboids) are fused with other prerecorded sensors (blue cuboids) and send to the rendering; 5. Virtual environment is generated in realtime using Odysseus Studio and virtual agent’s voice is computer generated using MacVoiceOver. Voice was send to the participant through headphones.

The experiment was completed in two visits. Visit 1 involved completing informed consent, clinical interviews and questionnaires. Visit 2 occurred approximately 2 days later. It consisted on a virtual mimicry experiment. We designed an interactive protocol comprising two conditions, a mimicry and a no-mimicry condition, where participants stood up in front a photo-realistic 3D virtual agent displayed on a large TV screen (Fig. 2). During the interaction and the two experimental conditions, the virtual agent provided information about some healthy issues: physical activity levels, quality of diet and sleep and how to quit smoking. Texts were vocally delivered by the virtual agent using a computer-generated voice and were synchronized with the virtual agent motion. The motion of the virtual agent was pre-recorded by an actor whose upper-body movements (head, torso, arms and forearms) were tracked with 3D Inertial Measurement Units (IMU). In the mimicry condition, the virtual agent mimics the participants’ head and torso movements with a delay varying randomly between 0.5 s and 4 s, while in the no-mimicry condition, head and torso movements were pre-recorded. Every 5 s to 15 s, the virtual agent was scratching his arm to induce behavioral mimicry by the participant. During the interaction, participants had their movements recorded by six IMUs attached to their arms, forearms, torso and head (Fig. 3). One message was delivered per condition. Each condition lasted 300 s. Conditions were fully counterbalanced across participants. We measured in each condition the range of motion performed by the arms of the participant and the degree of behavioral synchronization between the virtual agent and the participant using the *Forearms imitation motion*.

### Data Analysis

We measured the degree of synchronization behavior between the virtual agent and the participant using the *Forearms imitation motion*. 2 sets of 6 sensors (inertial measurement units) were used in this experiment. The first set corresponded to the virtual agent motion and the second set to the participant motion. Each sensor data called quaternion was recorded at a sampling frequency of 50 Hz (±1 Hz). Quaternions are simpler and more efficient representations of a rotation in a 3D space than Euler angles. Raw quaternions were normalized to ensure the robustness of further computations^57^. Normalized quaternions were then interpolated on a constant 50 Hz sampling rate using the Spherical Linear Quaternion Interpolation SLERP method^58^. We computed the amount of rotation between two samples using the natural metric for the rotation group (induced by the shortest path between its two elements); specifically we used its functional form based on the inner product of unit quaternions, which is most computationally efficient^59^. The amount of rotation was therefore unwrapped to avoid 2pi jumps in order to allow the next step of the analysis. Cross wavelet transform between the amount of rotation of the forearms of the participant and the virtual agent were computed^59^ giving rise to 3 time-frequency representations (Left forearm of the virtual agent versus Left forearm of the participant, Left forearm of the virtual agent versus Right forearm of the participant, and Right forearm of the virtual agent versus Right forearm of the participant). Cross wavelet transforms indicates the amount of synchronization between two time series for each sample in time and each period interval. Cross wavelet transforms were computed only in the range of period between 0.5 s to 8 s, corresponding to the range of behavioral mimicry usually described in the literature^60^. Significant areas (>0.95) of each of these 3 time-frequency representations were extracted and superimposed. Significant areas can be interpreted as synchronization levels significantly higher than random noise. The forearm imitation motion was finally calculated as a percentage of the trial where significant relationships between a movement of the virtual agent forearms and a movement of the participant forearms was detected.

Considering the statistical analysis, group differences and interactions were analysed with ANOVA tests. Post-hoc tests were used when the nature of the effects had to be specified. Size effects on our repeated ANOVA design have been reported using the partial eta squared^61^ η_p_^2^, and interpreted following Cohen^62^ where 0.02 corresponds to a small effect, 0.13 to a medium effect and 0.26 to a large effect. When necessary, alpha value of significance was corrected using the Bonferroni procedure.

## Electronic supplementary material

Supplementary Materials
