# Supplementary material for: Using mimicry of body movements by a virtual agent to increase synchronization behavior and rapport in individuals with schizophrenia
Source: Sci Rep. 2018 Nov 26;8:17356. doi: 10.1038/s41598-018-35813-6 (PMC6255843; doi:10.1038/s41598-018-35813-6)
Supplement: Supplementary file 1 — Supplementary Materials [file 41598_2018_35813_MOESM1_ESM.pdf]

Supplementary materials: Using mimicry of body movements by a virtual agent to increase synchronization behavior and rapport in individuals with schizophrenia

Stéphane Raffard[1,2,*], Robin N. Salesse[1,3,*], Catherine Bortolon[1,2], Benoit G. Bardy[3,4], José Henriques[5], Ludovic Marin[3], Didier Stricker[5], and Delphine Capdevielle[1,6]

[1]University Department of Adult Psychiatry, Hôpital de la Colombière, CHU Montpellier, Montpellier, France
[2]Epsylon Laboratory EA 4556, Univ Paul Valéry Montpellier 3, Univ Montpellier, Montpellier, France
[3]EuroMov, Univ. Montpellier, Montpellier, France
[4]Institut Universitaire de France, Paris, France
[5]German Research Center for Artificial Intelligence (DFKI GmbH), University of Kaiserslautern, Allemagne
[6]INSERM U-1061, Montpellier, France
[*]To whom correspondence should be addressed; Laboratory Epsylon, EA 4556, Montpellier University 3, University Department of Adult Psychiatry, 39 Avenue Charles Flahault 34295 Montpellier Cedex 5, France. Tel.:+33 4 67 33 97 02; fax: +33 4 67 33 96 60 / Email: s-raffard@chu-montpellier.fr; salesse.robin@gmail.com

ABSTRACT

These supplementary materials give further information about the methodology used in this study.

Methodology

We proposed an experimental task of social interaction with avatars (Figure 1). Note that all participants provided an informed consent for publication of identifying information/images in an online open-access publication.

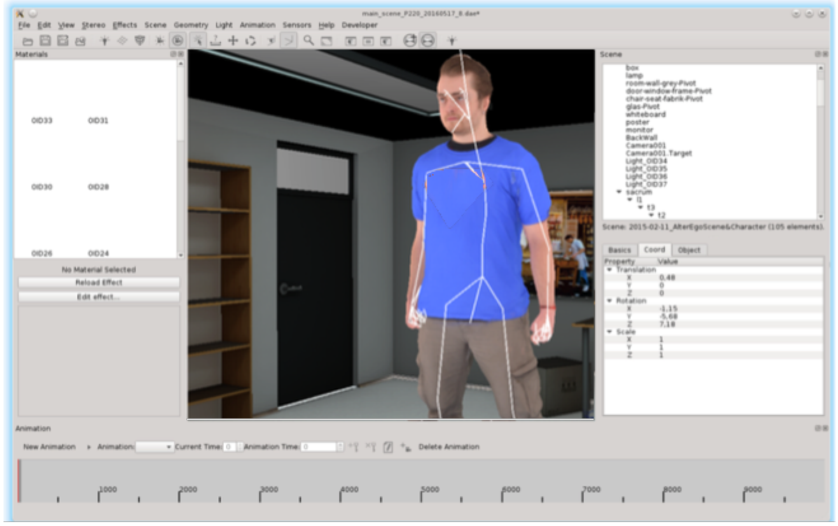

Figure 1. Representation of an avatar and its skeleton in its Virtual environment.

Avatars were created using real humans (one male and one female confederates). Their bodies were photographed in order to create their own 3D avatar. To do so, a system composed by 43 DSLR cameras (Nikon D3200, Canon EOS 100D, Canon EOS 500D) was used (Figure 2).

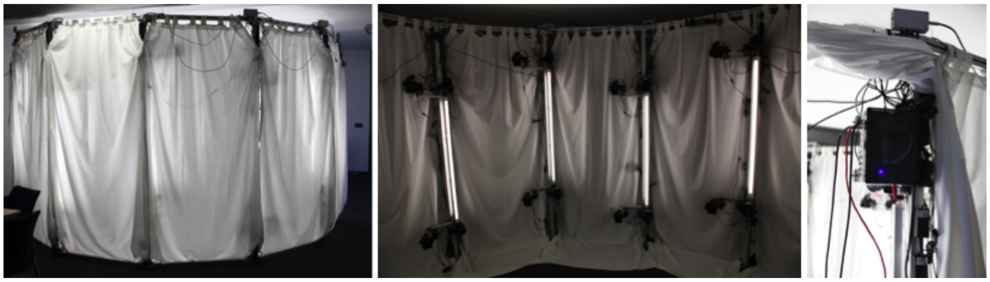

Figure 2. Pictures of the apparatus used to create the 3D avatars (CYBOS : CYlindar BOdy Scanner).

All cameras were synchronized using a hardware trigger controlled by a Raspberry PI. A pipeline was therefore used to create the 3D animated avatar ([1],[2]). Participants performed the experimental task of interaction, which includes two different conditions varying the behavioral (mimicry or no mimicry) similarity. In the mimicry condition, the avatar mimics the participants' head and torso movements, while in the no mimicry condition head and torso movements were pre-recorded. During the interaction, the avatar provided information about some healthy issues as well as some suggestions on how to improve physical activity levels, quality of diet, quality of sleep and how to quit smoking. All the different conditions were counterbalanced. Participants had their movements recorded by six sensors attached to their arms, forearms, torso and head (Figure 3).

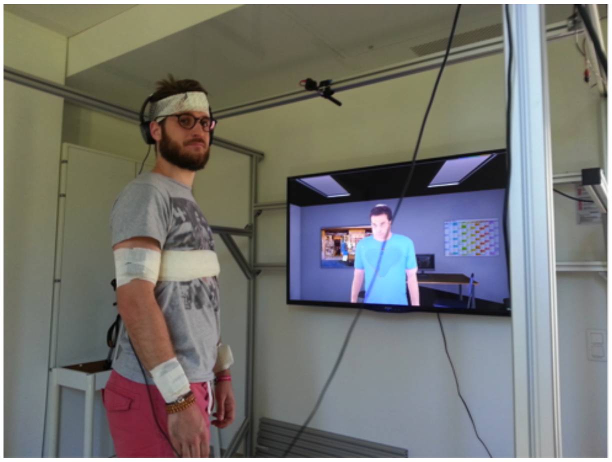

Figure 3. Picture showing the location of the 6 sensors (head, torso, both arms and forearms).

Between each condition, participants completed a questionnaire evaluating different aspects of the social interaction: "I felt comfortable while interacting with this avatar", "I think this avatar is attractive", "I like this avatar", and "I want to interact with this avatar again in the future". The questions were answered using a scale from -3 to 3, being -3 equals to "I do not agree at all", 0 "more or less", and 3 "I totally agree".

Protocol

Participants were asked to interact with an unknown photorealistic 3D avatar. Two models of avatars were used as a function of the gender of the participant (Ludo for males and Mia for females). The avatar was displayed following two mimicry conditions. In the no-mimicry condition, forearms, arms, torso and head of the avatar were displayed according to a prerecorded motion corresponding to the message being presented by the avatar. In the mimicry condition, whereas forearms and arms of the avatars followed the prerecorded motion, head and torso motions were actually the head and torso movements performed by the participant during the interaction with a randomly variable delay between .5 and 4s.

Set-up and procedure

Each participant wore a set of six sensors located on their forearms, arms, torso and head (Figure 4).

Each set was composed by a hacked TRIVISIO Colibri Wireless system with one USB-Dongle receiver and 6 Inertial Measurement Units. Data were sampled at a frequency of 100Hz with a spatial resolution of 0.5° in all 3D axis since this system measure orientations/rotations and not displacements in the Cartesian space ([3]) Participants have to interact in each of

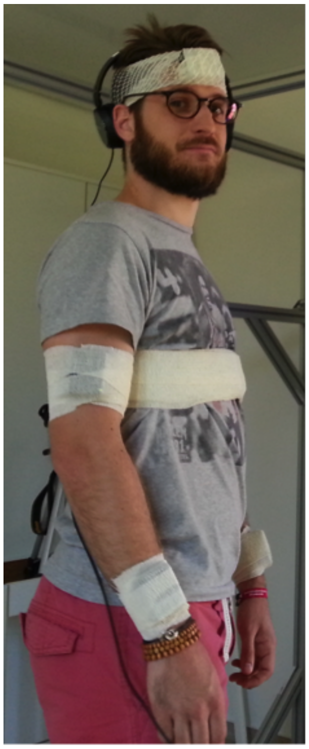

Figure 4. Close-up on the Sensors location.

the 2 conditions (Mimicry ; No-mimicry). During each condition, one of four message dealing either with Having a good Sleep, or Practicing physical activity, Having an healthy food or Quit smoking was said by the avatar to the participant through headphones. The four messages were counterbalanced across conditions and participants. Each condition was split in 2 parts. The first part corresponded to an introduction of the avatar telling his/her name, asking for the name of the participant who had to answer, and finally an invitation to discuss a subject. Whatever the condition, the motion of the avatar was prerecorded for the whole body during this part. In the second part, the avatar was talking about one of the four messages and his motion depended on the mimicry condition (Figure 5).

Moreover, every roughly 15 seconds, the avatar was performing specific movements such as arm scratching, shoulder lifting, or neck relaxation in order to eventually induce a synchronization behavior from the participant.

Dependent variables

Several information was extracted from the sensors. Each sensor provides a quaternion (a super complex number: ai+bj+ck+d) giving the 3D orientation of each segment with respect to the neutral pose (Figure 1). Considering that the orientation of the segments of both the avatar and the participant were recorded simultaneously, two kinds of dependent variables were extracted. First, DVs corresponding to the motion of the participant himself and second, DVs corresponding to the coordination/mimicry between the participant and the avatar. We therefore computed the global amount of movement for the sum of arms sensors (cf. Energy Motion Analysis for an equivalent 2D like procedure). We also computed the maximum of the cross-covariance between the time series of the avatar versus the ones of the participant to estimate the amount of mimicry. This last variable was also computed for the sum of arms sensors.

Methods and Data analysis

We measured the degree of synchronization between the avatar and the participant using the Forearms imitation motion. 2 sets of 6 sensors (inertial measurement units) were used in this experiment. The first set corresponded to the avatar motion and the second set to the participant motion. Each sensor data called quaternion was recorded at a downsampled frequency of 50 Hz ($\pm$ 1 Hz). Quaternions are simpler and more efficient representations of a rotation in a 3D space than Euler angles. Raw quaternions were normalized to ensure the robustness of further computations ([4]). Normalized quaternions were then interpolated on a constant 50Hz sampling rate using the Spherical Linear Quaternion Interpolation SLERP method ([5]). We computed the amount of rotation between two samples using the natural metric for the rotation group (induced by the shortest path between its two elements); specifically we used its functional form based on the inner product of unit quaternions, which

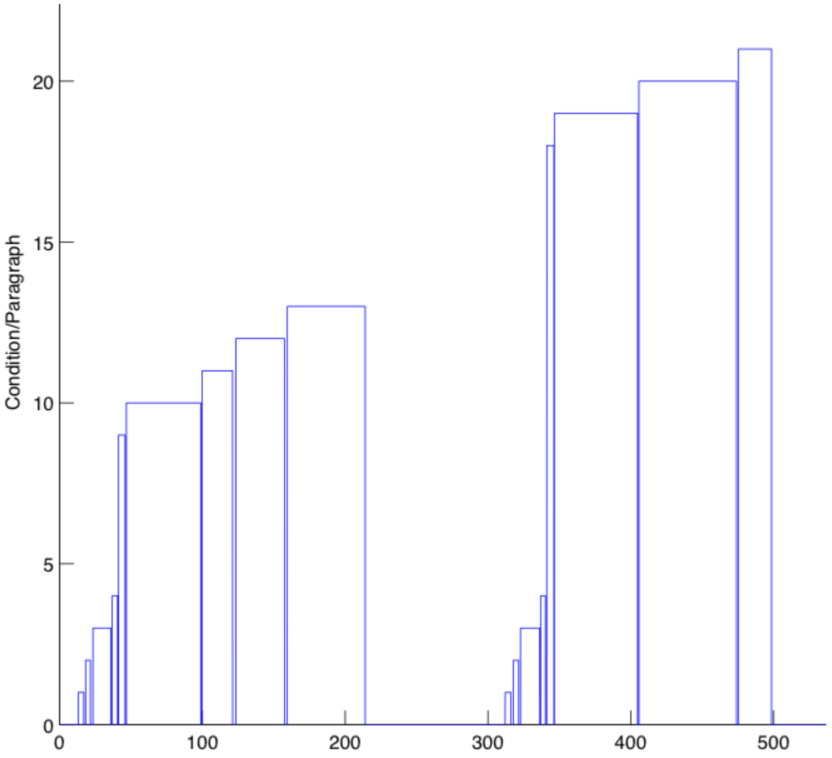

Figure 5. Example of a full session. Numbers in ordinate corresponds to the paragraph said by the avatar and numbers in abscises corresponds to the time in seconds.

is most computationally efficient ([6]). $amountOfRotation = \cos^{-1}(a_t \times a_{t+1} + b_t \times b_{t+1} + c_t \times c_{t+1} + d_t \times d_{t+1})$ where a, b, c and d correspond to the real numbers of the algebric representation of a quaternion a + bi + cj + dk, and i, j and k are the fundamental quaternion units. The $amountOfRotation$ was therefore unwrapped to avoid 2pi jumps in order to allow the next step of the analysis. Cross wavelet transform between the $amountOfRotation$ of the forearms of the participant and the avatar were computed ([7]) giving rise to 3 time-frequency representations (Left forearm of the avatar versus Left forearm of the participant, Left forearm of the avatar versus Right forearm of the participant, and Right forearm of the avatar versus Right forearm of the participant). Cross wavelet transforms were computed only in the range of period between .5s to 8s, corresponding to the range of automatic imitation usually described in the literature ([8]). Significant areas (>.95) of each of these 3 time-frequency representations were extracted and superimposed. The forearm imitation motion was finally calculated as a percentage of the trial where significant relationships between a movement of the avatar forearms and a movement of the participant forearms was detected (Figure 6).

References

1. Golyanik, V., Reis, G., Taetz, B. & Strieker, D. A framework for an accurate point cloud based registration of full 3d human body scans. In *2017 Fifteenth IAPR International Conference on Machine Vision Applications (MVA)*, 67–72, DOI: 10.23919/MVA.2017.7986778 (2017).

2. Wasenmüller, O., Peters, J. C., Golyanik, V. & Stricker, D. Precise and automatic anthropometric measurement extraction using template registration. In *Proceedings of the 6th International Conference on 3D Body Scanning Technologies, Lugano, Switzerland, 27-28 October 2015*, 155–160, DOI: 10.15221/15.155 (2015). Exported from https://app.dimensions.ai on 2018/09/14.

3. Filippeschi, A. *et al.* Survey of motion tracking methods based on inertial sensors: A focus on upper limb human motion. *Sensors* **17** (2017).

4. Stevens, B. L., Lewis, F. L. & Johnson, E. N. *Aircraft control and simulation: dynamics, controls design, and autonomous systems* (John Wiley & Sons, 2015).

5. Dam, E. B., Koch, M. & Lillholm, M. *Quaternions, interpolation and animation*, vol. 2 (Citeseer, 1998).

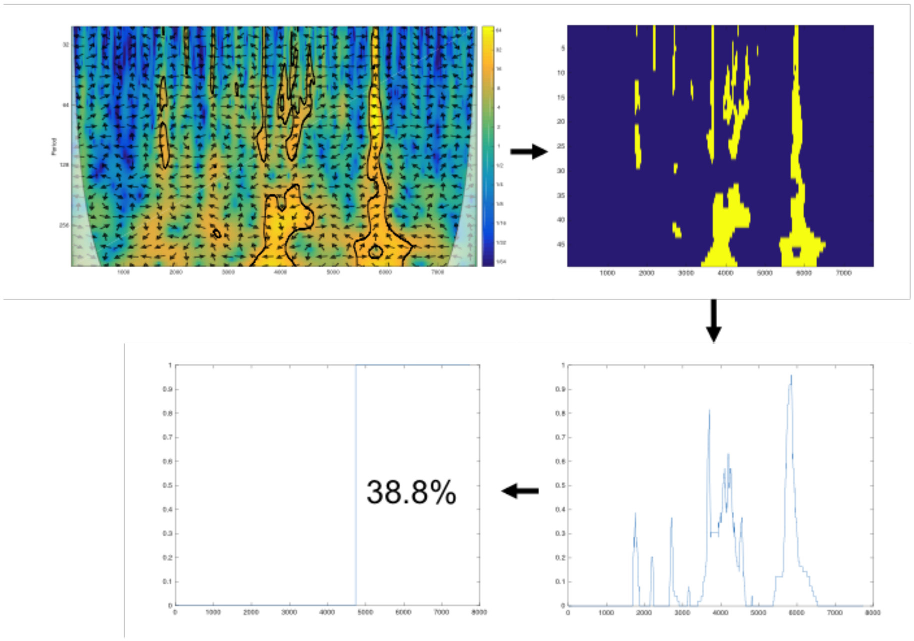

Figure 6. Representation of the significant steps of the analysis.

6. Huggins, D. J. Comparing distance metrics for rotation using the k-nearest neighbors algorithm for entropy estimation. *J. computational chemistry* **35**, 377–385 (2014).

7. Grinsted, A., Moore, J. C. & Jevrejeva, S. Application of the cross wavelet transform and wavelet coherence to geophysical time series. *Nonlinear processes geophysics* **11**, 561–566 (2004).

8. Eaves, D. L., Turgeon, M. & Vogt, S. Automatic imitation in rhythmical actions: kinematic fidelity and the effects of compatibility, delay, and visual monitoring. *PLoS One* **7**, e46728 (2012).
